# Whole Genome Sequence of *Dermacoccus* *abyssi* MT1.1 Isolated from the Challenger Deep of the Mariana Trench Reveals Phenazine Biosynthesis Locus and Environmental Adaptation Factors

**DOI:** 10.3390/md18030131

**Published:** 2020-02-25

**Authors:** Wael M. Abdel-Mageed, Bertalan Juhasz, Burhan Lehri, Ali S. Alqahtani, Imen Nouioui, Dawrin Pech-Puch, Jioji N. Tabudravu, Michael Goodfellow, Jaime Rodríguez, Marcel Jaspars, Andrey V. Karlyshev

**Affiliations:** 1 Department of Pharmacognosy, College of Pharmacy, King Saud University, P.O. Box 2457, Riyadh 11451, Saudi Arabia; alalqahtani@ksu.edu.sa; 2 Department of Pharmacognosy, Faculty of Pharmacy, Assiut University, Assiut 71526, Egypt; 3 Marine Biodiscovery Centre, Department of Chemistry, University of Aberdeen, Old Aberdeen, Scotland, AB24 3UE, UK; r01bj16@abdn.ac.uk; 4 School of Life Sciences Pharmacy and Chemistry, Faculty of Science, Engineering and Computing, Kingston University, Kingston upon Thames, Penrhyn Road, KT1 2EE, UK; b.lehri@hotmail.co.uk (B.L.); a.karlyshev@kingston.ac.uk (A.V.K.); 5 School of Natural and Environmental Sciences, Newcastle University, Newcastle upon Tyne, NE1 7RU, UK; Imen.Nouioui@newcastle.ac.uk (I.N.); Michael.Goodfellow@newcastle.ac.uk (M.G.); 6 Centro de Investigacións Científicas Avanzadas (CICA) e Departamento de Química, Facultade de Ciencias, Universidade da Coruña, 15071 A Coruña, Spain; dawrin.j.pech@udc.es (D.P.-P.); jaime.rodriguez@udc.es (J.R.); 7 School of Forensic and Applied Sciences, Faculty of Science and Technology, University of Central Lancashire, PR1 2HE, Preston, UK; jtabudravu@uclan.ac.uk

**Keywords:** Mariana Trench, *Dermacoccus abyssi* MT1.1^T^, dermacozines, genome sequencing, biosynthetic gene clusters

## Abstract

*Dermacoccus abyssi* strain MT1.1^T^ is a piezotolerant actinobacterium that was isolated from Mariana Trench sediment collected at a depth of 10898 m. The organism was found to produce ten dermacozines (A‒J) that belonged to a new phenazine family and which displayed various biological activities such as radical scavenging and cytotoxicity. Here, we report on the isolation and identification of a new dermacozine compound, dermacozine M, the chemical structure of which was determined using 1D and 2D-NMR, and high resolution MS. A whole genome sequence of the strain contained six secondary metabolite-biosynthetic gene clusters (BGCs), including one responsible for the biosynthesis of a family of phenazine compounds. A pathway leading to the biosynthesis of dermacozines is proposed. Bioinformatic analyses of key stress-related genes provide an insight into how the organism adapted to the environmental conditions that prevail in the deep-sea.

## 1. Introduction

Recently, the search for new natural products that can be developed as resources for healthcare has been focused on the isolation of micro-organisms from extreme biomes on the premise that harsh environmental conditions give rise to unique diversity which in turn is a source of novel chemistry [1,2]. Culture-dependent strategies show that taxonomically novel actinobacteria isolated from deep-sea sediments and hyper-arid desert soils are a remarkably good source of new bioactive natural products [2,3,4]. A case in point is *Dermacoccus abyssi* MT1.1^T^, a novel piezotolerant strain, isolated from sediment collected from the Challenger Deep of the Mariana Trench in the Pacific Ocean [5], which produces a new ten-membered family of phenazines, the dermacozines [6,7]. *Dermacoccus abyssi* MT1.1^T^ (family *Dermacoccaceae*, order *Micrococcales*), is a Gram-positive actinobacterium, which forms coccoid cells (diameter 0.8–1.5 μm) that occur in irregular clusters. It grows well on glucose-yeast extract agar (GYEA) forming cream to pale-yellow, circular, convex, smooth, glistening colonies after 5 days of incubation at temperatures between 10 and 37 °C, with optimal growth at 28 °C. It grows well at 40 MPa as well as at normal atmospheric pressure in glucose-yeast extract broth [8]. Extremotolerant and extremophilic actinobacteria are in great demand in the search for new secondary metabolites for drug discovery using state-of-the-art technologies, notably genome mining [9].

**Figure 1:**
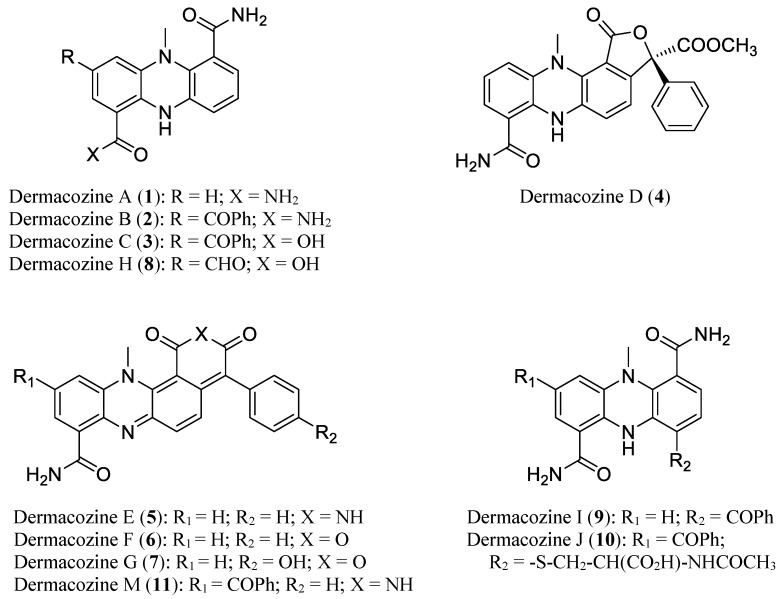
Chemical structures of *D. abyssi* dermacozines (**1**–**11**).

Phenazines belong to a large class of nitrogen-containing heterocyclic redox-active pigments of bacterial origin. They are produced by Gram-negative bacteria (e.g., *Pseudomonas*), and Gram-positive bacteria (e.g., *Streptomyces*) and by archaeal *Methanosarcina* species, and exhibit antibiotic, antitumor, insecticidal, and antiparasitic activities [10,11,12]. The core structure of phenazines consists of a pyrazine ring (1,4-diazobenzene) showing two annulated benzenes. Several phenazines, the dermacozines, are produced by *D. abyssi* MT1.1^T^ and MT1.2 (Figure 1). These are highly pigmented compounds belonging to a class of reduced phenazine-based marine products which contain a *N*-methyl bridge connecting two or more fused benzene rings [6,13]. Ten members of this family, dermacozines A–J, were identified and found to display various biological activities, such as radical scavenging and cytotoxicity against the leukemia cell line K562. Our ongoing studies on *D. abyssi* MT1.1^T^ resulted in the detection and characterization of an additional compound, dermacozine M. In this report, we describe the isolation and structural determination of this new dermacozine. In addition, the generation of a whole genome sequence of *D. abyssi* MT1.1^T^ led to the discovery of the phenazine biosynthetic gene cluster and the identification of candidate genes necessary for bacterial adaptation to extreme environmental conditions that prevail in the deep sea.

## 2. Results and Discussion

### 2.1. Compound Identification

Dermacozine M (**11**) was isolated as a navy-blue powder. HRESIMS showed pseudomolecular ion peaks at *m/z* 501.1550 [M + H]^+^, 523.1364 [M + Na]^+^, 1001.3031 [2M + H]^+^, and 1023.2848 [2M + Na]^+^ consistent with a molecular formula of C_30_H_20_O_4_N_4_, Δ = −1.4 ppm, which requires 23 degrees of unsaturation. The UV/Vis spectrum showed absorption maxima at 590, 380, 285, and 245 nm.

**Figure 2:**
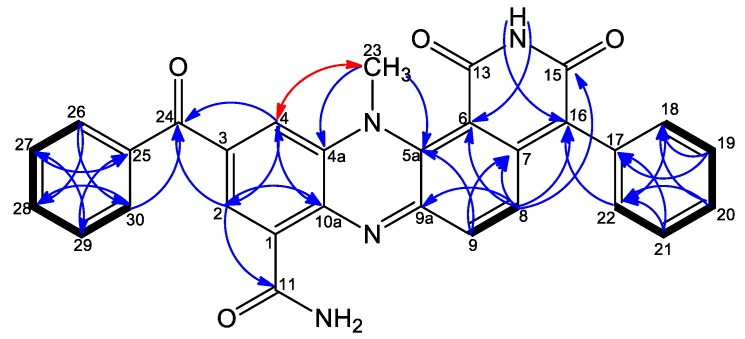
Selected COSY (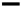), NOESY (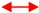), and HMBC (H
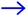
C) correlations.

^13^C NMR shifts for compound **11** obtained from overlaid HMBC and ^1^H-^13^C HSQC spectra exhibited one methyl signal at *δ_H_* 3.69 (CH_3_-23), fourteen aromatic methines grouped by ^1^H-^1^H COSY into four spin systems (Figure 2) comprising H-2, H-4 [*δ_H_* 8.21 (d, 1.8), 8.13 (d, 1.8)]; H-8, H-9 [*δ_H_* 7.25 (d, 9.7), 7.35 (d, 9.7)]; and two monosubstituted benzene rings H-18 – H-22 [*δ_H_* 7.33 (2H, dd, 7.5, 1.3), 7.49 (2H, t, 7.5), 7.44 (1H, td, 7.5, 1.3)], and H-26 – H-30 [*δ_H_* 7.85 (2H, dd, 7.5, 1.5), 7.64 (2H, dd, 7.5, 1.5), 7.76 (1H, m)] (Table 1). Additionally, fourteen quaternary carbon atoms were identified from ^1^H-^13^C HSQC and ^1^H-^13^C HMBC as well as ^1^H-^13^C HSQC and a 2 Hz long range ^1^H-^13^C HMBC spectra, including three carbonyls C-11, C-15, and C-24 (*δ_C_* 165.5, 161.7, and 194.4); no correlation was observed either to/or from the C-13 carbonyl. Comparison with previously published dermacozines and simulation with the ACD Lab neural network algorithm showed that the C-13 chemical shift was predicted to appear at *δ_C_* 161.9. Moreover, four carbons were identified next to nitrogen atoms C-4a, C-5a, C-9a, and C-10a (*δ_C_* 134.1, 139.2, 152.3, and 137.4); six olefinic carbons C-1, C-6, C-7, C-16, C-17, and C-25 (*δ_C_* 131.0, 100.8, 139.4, 123.4, 133.9, and 136.5) were also detected. Furthermore, HMBC correlations were not observed with C-3; chemical shift simulation with ACD lab software identified the chemical shift of C-3 at *δ_C_* 134.9, which is compatible with data for previously published dermacozines [6,13].

The ^1^H–^13^C HSQC NMR spectrum showed that three protons which lacked correlation to carbons were identified as one NH_2_ group [NH_2_-12 (*δ_H_* 7.99, 8.93)]; and one NH group carboxylic imide moiety [NH-14 (*δ_H_* 11.34)]. The connectivity between the spin systems was established from key ^1^H-^13^C HMBC correlations, including correlations from H-2 to C-4/C-11; from H-4 to C-2; from H-2/H-4 to C10a/C-24, thereby clearly defining the positions of the carboxamide group and the benzoyl moiety on C-1 and C-3, respectively. Furthermore, correlations from the methyl group CH_3_-23 to C-4a and C-5a accompanied with NOESY correlation with H-4 (Figure 2) confirmed its position relative to C-4a and C-5a. HMBC correlations from H-8 to C-6/C-9a together with a correlation from H-9 to C-7 confirmed the positions of C-6, C-7, and C-9a relative to the H-8, H-9 spin system. Finally, observed HMBC correlations of H-8, H-18/H-22 to C-16 confirmed the position of a monosubstituted benzene ring at C-16. Accordingly, the structure of **11** was identified as 3- benzoyl dermacozine E [6], the latter was named dermacozine M.

**Figure 3:**
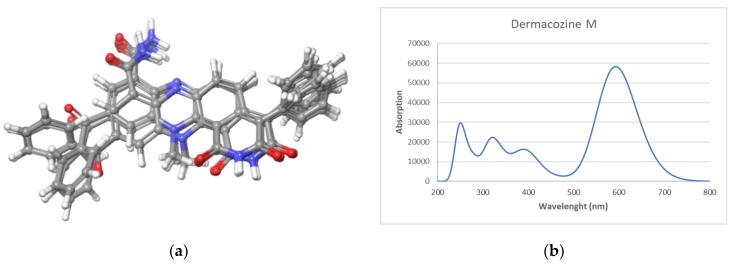
Dermacozine M: (**a**) Conformers obtained from the conformational search; (**b**) Theoretical UV spectra.

The calculated UV spectrum was simulated using a time-dependent DFT (TDDFT) calculation. Dermacozine M was initially submitted to a conformational search performed in the Macromodel module implemented in Maestro Quantum mechanical software (Schrödinger). Sixteen conformers were found within a 3.5 kcal/mol energy threshold from a global minimum. All these conformers were geometrically optimized, and frequency calculated by using a density functional theory (DFT) method at the HSEH1PBE/cc-pVDZ level (see computational details in the experimental section). In all calculations, the ethanol environment was simulated using the Polarizable Continuum Model, (PCM) by the integral equation formalism variant (IEFPPCM) as is implemented in Gaussian 09. The resulting UV spectra were combined by Boltzmann weighting to give a composite spectrum [14,15,16]. The spectra showed an UV/vis maximum at 592.6 nm (Figure 3), a result in perfect agreement with the experimental data.

### 2.2. Genome Sequencing and Annotation

The whole genome sequencing reads of *D. abyssi* MT1.1^T^ were generated by using an Ion Torrent PGM instrument, 316v2 chips, and a 400-bp sequencing kit. Assembly of the reads with the Torrent SPAdes plugin (v. 3.1.0) and CLC Genomics Workbench de novo assembly programs resulted in 55 contigs over 1 kb and up to 340.6 kb in size. The total size of the genome assembly was 3,160,906 bp with 122.46 × fold coverage and a 68.12% G + C content. The size and G + C content of the studied strain are in a good agreement with those of *D. abyssi* HZAU 226, the only other member of the species for which a full genome sequence is available.

The genome sequence was annotated using the NCBI prokaryotic genome annotation pipeline [17,18,19], as well as RAST [16], which predicted 3335 genes, 2959 CDSs, 163 pseudogenes, 6 rRNAs, 49 tRNA, 3 ncRNA, and 82 frameshifted genes within the 55 contigs in *D. abyssi* MT1.1^T^ (Table 2).

### 2.3. Secondary Metabolite-Biosynthetic Gene Clusters

The draft genome of *D. abyssi* MT1.1^T^ was examined for the presence of secondary metabolite-biosynthetic gene clusters using antiSMASH 5.1.0 [20]. The results showed the presence of a nonribosomal peptide synthetase (NRPS) in addition to five potential secondary metabolite gene clusters responsible for the biosynthesis of ectoine, siderophores, a bacteriocin, terpenes, and a phenazine.

#### Phenazine Biosynthetic Gene Clusters

**Figure 4:**
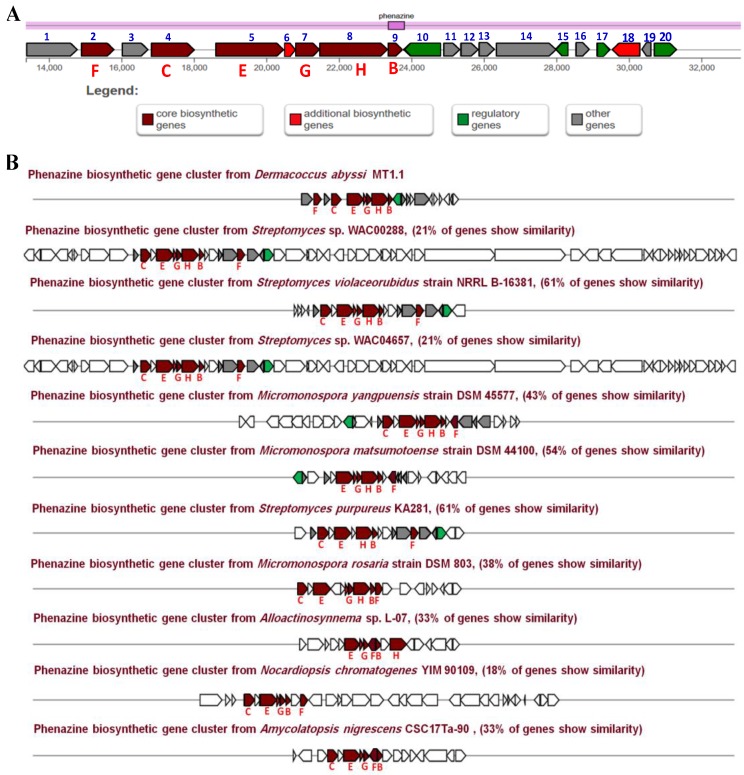
(**A**) Genetic organization of a putative phenazine gene cluster present in the genome of *D. abyssi* MT1.1^T^; (**B**) Comparison of the putative phenazine gene clusters from *D. abyssi* MT1.1^T^ with characterized homologous gene clusters found in the genome of other bacteria.

A putative phenazine biosynthetic gene cluster (Phz BGC) responsible for the biosynthesis of dermacozines was identified in the genome of *D. abyssi* MT1.1^T^. The analysis of this cluster revealed the presence of twenty genes involved in the dermacozine biosynthetic pathway, which belonged to four main functional groups, including core, additional, regulatory, and other biosynthetic genes of various catalytic functions (Figure 4). The predicted functions of the genes are listed in Table 3.

**Figure 5:**
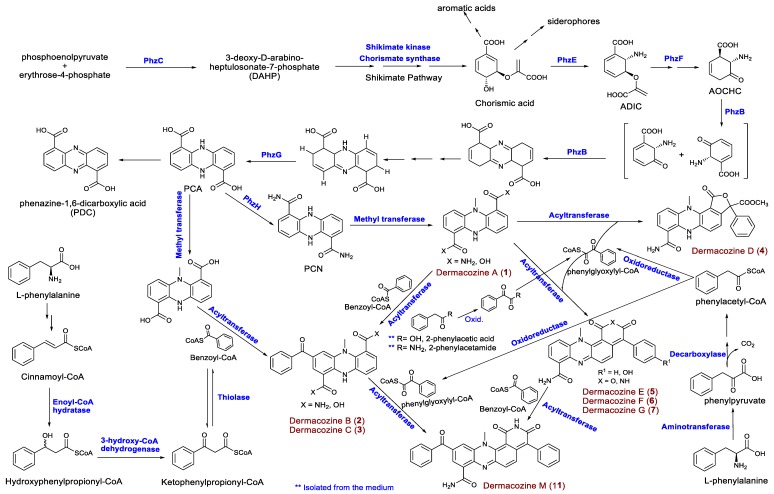
Proposed biosynthetic pathways for the synthesis of dermacozines by *D. abyssi* MT1.1^T^.

Dermacozine biosynthesis, like that of most phenazine compounds, starts via the shikimate pathway. Shikimic acid is converted to chorismic acid that is subsequently transformed to 2-amino-2-deoxyisochorismic acid (ADIC) necessary for the formation of the core structure of phenazines (Figure 5). Six genes comprising the *phzBCEFGH* cluster are involved in the biosynthesis of dermacozines. PhzE and PhzF probably catalyze the sequential formation of 2-amino-2-desoxyisochorismic acid (ADIC) and 6-amino-5-oxocyclohex-2-ene-1-carboxylic acid (AOCHC) [21]; PhzB and -G are believed to be involved in the oxidation/aromatization of initial tricyclic species [22]. Furthermore, PhzH (asparagine synthase) catalyzes transamidation leading to the formation of 5,10-dihydrophenazine-1,6-dicarboxamide (PCN) from 5,10-dihydrophenazine-1,6-dicarboxylic acid (PCA) [23]. Additional biosynthetic genes, such as those encoding an oxidoreductase thio-amide protein, as well as alpha/beta hydrolase with an esterase function and a group of regulatory proteins, were also detected.

Additional analysis of the *D. abyssi* MT1.1^T^ genome revealed the presence of genes encoding additional proteins that might be involved in the biosynthesis of dermacozines, including shikimate kinase, dehydroquinate synthase, and chorismate synthase for shikimic acid biosynthesis; aminotransferase, enoyl-CoA hydratase, 3-hydroxyacyl-CoA dehydrogenase, and thiolase for benzoyl-CoA biosynthesis; acyltransferase that putatively catalyzes the condensation of benzoyl-CoA with the tricyclic phenazine structure (Table 4).

### 2.4. Genomic Insights Into Adaptation Strategies

The Mariana Trench system is a non-accretionary convergent plate found between the Philippine Sea and subducting Pacific plates. The Mariana Trench is characterized by its elevated hydrostatic pressure (depth 10,915 to 10,920 m), low temperature (approx. ∼1 °C), high salinity, low oxygen, nitrate, nitrite, and phosphate concentrations and by low pH [6].

Stress associated genes reported in other bacteria were sought from the genome sequence of *D. abyssi* MT1.1^T^ to get insight into adaptation for such environments. A detailed discussion of the genes involved in stress responses is provided below.

#### 2.4.1. Cold Shock Response

Cold shock affects cell growth, the rates of DNA, RNA, and protein synthesis, as well as cell membrane structure and function in deep-sea habitats. *D. abyssi* MT1.1^T^ has genes encoding cold-shock proteins and cold-shock inducible proteins such as ClpB, GroL, GroES, and DEAD box RNA helicase (Table S1). The ability of deep-sea bacteria to produce a variety of cold shock inducible proteins is essential for low temperature adaptation of many deep-sea psychrotrophic bacteria, such as *Pseudomonas putida* and *Shewanella violacea* [24,25,26]. Furthermore, a gene encoding DEAD/DEAH-box RNA helicase is induced by cold shock and has been shown to be implicated in cellular processes such as ribosome biogenesis, translation initiation and termination, and cell growth and differentiation. At low temperature, DEAD/DEAH-box RNA helicases play important roles in cold shock response and cold adaptation by destabilizing RNA duplexes to promote translation initiation, and by participating in assembly of the 50S ribosomal subunit [27].

#### 2.4.2. Osmotic Stress Response

In deep-sea habitats, such as in the Mariana Trench, bacteria are exposed to high pressure conditions, which affect the extracellular environment leading to disturb internal osmotic balances. The accumulation of different small organic molecules (compatible solutes) is one of the ways for cells to readjust the balance of internal osmotic pressures. Mining of the *D. abyssi* MT1.1^T^ genome revealed the presence of many genes associated with the synthesis and accumulation of such solutes, including *betA*, *opuA* for choline, and glycine/betaine regulation and transport. Another predicted gene encodes the Aquaporin Z protein that mediates the transport of water molecules across cell membranes [28] (Table S1). The presence of these genes indicates the important role played by compatible solutes, such as osmoregulators, to overcome osmotic stress in deep sea environments.

*D. abyssi* MT1.1^T^ grows well at 40 MPa [8]. Mining of the *D. abyssi* genome revealed a number of genes required for pressure-sensing and pressure-adaptation in other piezotolerant and piezophilic bacteria. These genes encode terminal oxidases, such as aspartate β-D-semialdehyde dehydrogenase *asd*, cytochrome bd biosynthesis proteins *cydAB*, thiol reductant ABC exporter subunit *cydCD*, malate dehydrogenase *mdh*, and a single-stranded DNA binding protein *ssb* were discovered in piezotolerant/piezophilic bacteria such as *Photobacterium profundum* strain SS9 and *Shewanella* species [29] (Table S1).

In high pressure environments, the transport of compounds such as amino acid is reduced, leading to upregulation of certain transporters to counteract significant inhibition of amino acids uptake into cells at high pressure [30]. Different types of ABC transporter permeases were detected in the *D. abyssi* MT1.1^T^ genome including ABC transporter permease, an amino acid ABC transporter permease, and a branched-chain amino acid ABC transporter permease that is up-regulated at high pressure to compensate for the reduction of transportation functionality [31]. Another significant group of proteins involved in protein export across the outer membrane are the export proteins SecD and SecF. Strains lacking these proteins have been shown to be cold and high pressure-sensitive [31].

Many biological processes, such as membrane transport, intracellular signaling, metabolic electron transport, protein–protein interactions, and gene regulation, are dependent on the dynamic state and physical structure of membranes hence the maintenance of biological membranes in cold temperature and high pressure conditions is essential for bacterial growth and survival [32].

#### 2.4.3. Oxidative Stress Response

The *D. abyssi* MT1.1^T^ genome contained copies of antioxidant enzyme-encoding genes that counteract the effect of reactive oxygen species (ROS) generated at low temperatures, such as superoxide dismutase, catalase, thioredoxin, thioredoxin-disulfide reductase, peroxiredoxin, peroxiredoxin (OsmC family), NAD(P)H-quinone oxidoreductase, alkyl hydroperoxide reductase, glutathione S-transferase (family protein), arsenate reductase, and glutaredoxin (family proteins), all of which have the ability to repair damage caused to proteins and other cell components by ROS [31] (Table S2).

#### 2.4.4. Respiration

The *D. abyssi* MT1.1^T^ genome contains many genes encoding terminal oxidases for aerobic respiration, such as cytochrome *d* ubiquinol oxidase subunit I, II genes *cydAB*, and cytochrome *c* oxidase of the *aa_3_*-type (Table S2). Cytochrome *d* oxidase *cyd* exhibits a high affinity for O_2_, thereby facilitating respiration under microaerobic conditions, whereas *aa_3_*-type oxidase complexes show a low affinity for O_2_ and work only at high O_2_ tensions [24]. In addition, a number of terminal reductases (terminal electron acceptors) and distinct dehydrogenase encoding genes involved in respiratory chains were identified, such as those encoding arsenate reductase ArsC, ferredoxin reductase, as well as NADH-quinone oxidoreductase (NADH dehydrogenase-1) Nuo, glycerol-3-phosphate dehydrogenase, lactate dehydrogenase, and formate dehydrogenase. Moreover, multiple copies of genes encoding succinate dehydrogenases that can be used as electron donating systems under low oxygen conditions were detected in the genome of *D. abyssi* MT1.1^T^ (Table S2) [33]. This strain also has the capacity to produce many cytochrome oxidase complexes of different affinities for oxygen, implying that respiratory reductases and dehydrogenases allow the strain to adapt to different oxygen levels.

#### 2.4.5. Cell Wall/Membrane Alteration

Maintaining cell membranes in a liquid crystalline state is considered to be one of the most crucial requirements for organisms living in cold environments. Psychrophilic bacteria tend to retain membrane fluidity and functionality by incorporating lower melting point branched chain and/or polyunsaturated fatty acids (PUFAs), such as such as docosahexaenoic acid (DHA) and eicosapentaenoic acid (EPA) in their membrane lipids. Interestingly, analysis of the genome of *D. abyssi* MT1.1^T^ revealed the presence of genes encoding groups of enzymes involved in the synthesis of long chain PUFAs, such as acyl-CoA desaturase, enoyl-ACP reductase, enoyl-CoA hydratase (ECH), 3-hydroxyacyl-CoA dehydrogenase, long-chain fatty acid-CoA ligase, acyl dehydratase, phosphopantetheinyl transferase, UDP-N-acetylglucosamine 1-carboxyvinyltransferase, 3-oxoacyl-ACP reductase (FabG), ketoacyl-ACP synthase (FabH), enoyl (FabI), as well as phytoene/squalene synthase that is linked to membrane fluidity at low temperatures in the psychrophilic bacteria [34,35,36] (Table S3).

#### 2.4.6. Carbon Starvation and Storage

In nutrient-limiting conditions, such as in deep-sea ecosystems, organisms need to store carbon. The genome of the *D. abyssi* MT1.1^T^ exhibited the presence of genes encoding the carbon starvation protein A CstA, in addition to the glycogen synthesis protein GlgA, the glycogen debranching enzyme GlgX, and glycogen/starch/alpha-glucan phosphorylase, which is responsible for the breakdown of these storage molecules. The genome also included multiple copies of genes encoding for carbon-nitrogen hydrolase and carbonic anhydrase proteins essential for efficient CO_2_ fixation [31,37,38] (Table S3).

#### 2.4.7. Remineralization of Organic Matter

Since there is a dearth of organic matter in deep-sea habitats, global thermohaline circulation plays a crucial role in transferring nutrients from primary production sources. Accordingly, deep-sea bacteria respond quickly to the presence of organic matter by producing enzymes that convert extracellular complex organic compounds to easily metabolized molecules.

The *D. abyssi* MT1.1^T^ genome included genes encoding extracellular enzymes responsible for remineralization of organic matter (Table S4), including a group of proteases and polysaccharases able to decompose proteinaceous and polysaccharide materials derived from phytoplankton and dead marine vertebrates that sink to deep-sea floors [39,40].

With increased depth in the ocean, the level of organic nitrogen in deep-sea habitats is decreased. Access of *D. abyssi* MT1.1^T^ to nitrogen might be facilitated by the expression of purine and allantoin utilization genes.

Interestingly, almost all the candidate genes necessary for bacterial adaptation to extreme environmental conditions relevant to this study (Tables S1–S4) are also present in published genome sequences of other *Dermacoccus* strains, namely *D. abyssi* HZAU266 and *D. nishinomiyaensis* NCTC11039T, which are not extremophilic. This suggests that the ability of the strain to live under stress conditions not only depends on the presence or absence of stress genes but also on their expression levels, which in turn are regulated by complicated metabolic processes that may differ between organisms. Consequently, molecular mechanisms which determine how organisms cope with environmental conditions that prevail in the deep-sea habitat are worthy of further study.

## 3. Materials and Methods

### 3.1. General Experimental Procedures

NMR spectra were recorded in DMSO-*d_6_* (δ_C_ 39.5, δ_H_ 2.50) on a Bruker 600 MHz NMR spectrometer AVANCE III HD (Billerica, MA, USA) operating with a liquid N_2_ cooled ‘Prodigy’ cryoprobe. High-resolution ESI-MS data were obtained using a Thermo Instruments MS system (LTQ XL/LTQ Orbitrap Discovery, Thermo Scientific, San Jose, CA, USA) coupled to a Thermo Instruments HPLC system (Accela PDA detector, Accela PDA autosampler and Accela pump). HPLC separations were carried out using a Sunfire reversed phase column (C_18_, 5 µm, 250 × 10 mm) and an Agilent 1100 series gradient pump monitored using a DAD G1315 variable-wavelength UV detector.

### 3.2. Microorganism

*Dermacoccus abyssi* strain MT1.1^T^ was isolated from sediment collected from the Challenger Deep of the Mariana Trench (142°12ʹ372ʹʹ E; 11°19ʹ911ʹʹ N) at 10,898 m by using the remotely unmanned operated submarine “*Kaiko*”, during dive number 74, on 21/05/1998 [8].

### 3.3. Fermentation Conditions

For small-scale fermentation, a colony was scooped with a loop and seeding cultures prepared in 100 mL Erlenmeyer flasks with 25 mL GYE medium (yeast extract 4 g, d-glucose 4 g, MilliQ water 1 L adjusted to pH 7.0) after incubation for five days at 28 °C with agitation at 150 rpm.

For large-scale fermentation, six 2 L Erlenmeyer flasks, each of them containing 1000 mL of ISP2 medium (yeast extract 4 g, D-glucose 10 g, malt extract 10 g, MilliQ water 1 L) supplemented with 35 g/L ocean salt (H_2_Ocean + Pro Formula with trace elements, The Aquarium Solution Ltd., Hainault Industrial Estate, Ilford Essex, UK) was inoculated with 1.5 mL of the first stage seed culture and incubated at 28 °C with agitation at 150 rpm for 14 days.

### 3.4. Purification of Dermacozine M

Harvested fermentation broth from strain MT1.1^T^ (6 L) was mixed with 50 g/L Diaion HP20 resin (>250 um, Alfa Aesar, Ward Hill, MA, USA) for 24 h in a shaker at 28 °C and a setting of 150 rpm. The HP20 resin was eluted with methanol (3 × 500 mL) and then with acetone. Successive methanol and acetone extracts were combined and concentrated under reduced pressure, yielding the crude material (6758 mg) which was partitioned with liquid–liquid extraction (Kupchan method) [41]. The crude material was suspended in H_2_O and extracted with an equal volume of dichloromethane three times. The dichloromethane layer was dried then subsequently dissolved in 90% methanol and 10% H_2_O solution. This solution was subjected to extraction with an equal volume of *n*-hexane. The reminder of the methanol-H_2_O (90%/10%) solution, after *n*-hexane extraction, was adjusted to 50% methanol–50% H_2_O solution. This solution was extracted with an equal volume of dichloromethane three times. The dichloromethane fraction (379.9 mg) was subjected to standard silica gel (Fluorochem, 60A 40–63U MW = 60.083, Derbyshire, UK) column chromatography (Quickfit XA42 29/32, 19/26, 19 mm × 500 mm, Fisher Scientific, Loughborough, UK) with a 90% dichloromethane and 10% methanol mobile phase. The first visible colored band upon silica chromatography yielded a pure navy blue compound, namely dermacozine M (**11**) (2.8 mg).

Dermacozine M (**5**): bluish violet amorphous powder, 2.8 mg; UV: λ_max_^CH^_3_^OH+H^_2_^O^ 590, 380, 285, 245 nm; HRESIMS (*m*/*z* 501.1550 [M + H]^+^, 523.1364 [M + Na]^+^, 1001.3031 [2M + H]^+^, 1023.2848 [2M + Na]^+^, MF = C_30_H_20_N_4_O_4_, 500.1485, Δ = −1.4 ppm); ^1^H and ^13^C NMR data (DMSO-*d_6_*), see Table 1.

### 3.5. Computational Calculations

Conformational searches were performed by using the Macromodel module implemented in Maestro Quantum mechanical software [14]. The OPLS 2005 force field with methanol as solvent was used, and torsional enhanced sampling with 1000 or 10,000 steps was fixed using an energy window of 3.5 kcal/mol. Molecular geometry optimizations were performed at the DFT theoretical level using the Gaussian 09W package firstly with a B3LYP/6-31G(d) combination and then with HSEH1PBE/cc-pVDZ auto /IEFPCM model (ethanol as solvent) for energy and frequency calculations. After removing redundant conformers and those with imaginary frequencies, theoretical Boltzmann energy population-weighted UV was calculated by using PBEPBE/6-311++(3d,2p) with 24 states [15]. A graphical theoretical UV curve was obtained using the open software SpecDis V.1.71 (Berlin, Germany, 2017, https:/specdis-software.jimdo.com) [16].

### 3.6. Genome Sequencing Information

#### 3.6.1. DNA Extraction and Genome Sequencing

Genomic DNA was extracted from *D. abyssi* MT1.1^T^ following the modified CTAB method cited in current protocols in molecular biology [42]. A NEB Next Fast DNA Fragmentation and Library Preparation Kit (New England Biolabs) for Ion Torrent instruments was used to generate a sequencing library according to the manufacturer’s protocol. After enzymatic fragmentation, end repair and ligation to A1 and P2 adapters, followed by PCR amplification of 490–500 bp fragments were isolated and analyzed using the BioAnalyser 2100 and High Sensitivity DNA kit (Agilent Technologies LDA UK Limited, Cheshire, UK). The library was used for template preparation using the IonTorrent One Touch system and an Ion PGM Hi-Q™ View OT2 Kit, followed by recovery of positive Ion Sphere Particles using the One Touch ES enrichment system. The sequencing reaction was conducted using the IonTorrent PGM instrument, 316v2 chips and Ion PGM Hi-Q^TM^ View Sequencing Kit with 850 sequencing flows, according to manufacturer’s instructions (Life Technologies Limited, Paisley, UK).

#### 3.6.2. Detection of the Gene Clusters

The whole genome sequence of *D. abyssi* MT1.1^T^ was mined using antiSMASH 5.1.0 (“Antibiotic and Secondary Metabolites Analysis Shell”) [20], while the NCBI GenBank annotation pipeline and RAST were used for the detection of proteins and genes responsible for adaptation.

#### 3.6.3. Genbank Accession Number

This Whole Genome Shotgun sequence was deposited at DDBJ/ENA/GenBank under accession number QWLM00000000. The version described in this paper is version QWLM01000000.

## 4. Conclusions

*Dermacoccus abyssi* strain MT1.1^T^ is an example of a deep marine strain with a genome that harbors six uncharacterized secondary metabolite-biosynthetic gene clusters. The strain produces a unique family of phenazines, the dermacozines, including dermacozine M, which was extracted in the present study from a fermentation broth and its structure determined using NMR, high resolution MS, and by structural comparison with previously published dermacozines. Subsequently, data mining of the whole genome sequence of the strain lead to the detection of the phenazine BGC and genes involved in the biosynthesis of the unique dermacozines. The genome of *D. abyssi* strain MT1.1^T^ contained five secondary metabolite BGCs in addition to the phenazine one, the analysis of which may lead to the discovery of additional novel bioactive secondary metabolites. Bioinformatic analyses of the genome lead to the identification of putative stress-related genes that provided an insight into how the organism may cope with the environmental conditions that prevail in deep-sea habitats.

## Figures and Tables

**Table 1:** ^1^H (600 MHz) and ^13^C NMR (150 MHz) data (in DMSO-*d_6_*) for compound **11**.

no.	11	
	δ_C_, mult.	δ_H_, mult (*J* in Hz)
1	131.0, C	-
2	127.2, CH	8.21, d (1.8)
3	134.9^†^, C	-
4	119.8, CH	8.13, d (1.8)
4a	134.1, C	-
5a	139.2, C	-
6	100.8, C	-
7	139.4, C	-
8	130.1, CH	7.25, d (9.7)
9	134.8, CH	7.35, d (9.7)
9a	152.3, C	-
10a	137.4, C	-
11	165.5, C	-
12		a. 7.99, brs b. 8.93, brs
13	161.9 ^†^, C	-
14		11.34, brs
15	161.7, C	-
16	123.4, C	-
17	133.9, C	-
18	131.1, CH	7.33, dd (7.5, 1.3)
19	127.9, CH	7.49, t (7.5)
20	127.7, CH	7.44, td (7.5, 1.3)
21	127.9, CH	7.49, t (7.5)
22	131.1, CH	7.33, dd (7.5, 1.3)
23	45.6, CH_3_	3.69, s
24	194.4, C	-
25	136.5, C	-
26	129.6, CH	7.85, dd (7.5, 1.5)
27	128.6, CH	7.64, td (7.5, 1.5)
28	133.2, CH	7.76, m
29	128.6, CH	7.64, td (7.5, 1.5)
30	129.6, CH	7.85, dd (7.5, 1.5)

^†^ Shifts were not observed—calculated chemical shifts were made using the ACD Labs Neural Network algorithm.

**Table 2:** General features of the *D. abyssi* MT1.1 genome.

Features	*D. abyssi* (MT1.1)
Assembly size, bp	3,160,906
No. of contigs	55
G + C	68.1
Fold coverage	122.46×
Percentage of bases	99.7%
N50	159983
L50	7
Genes	3,335
CDs	2,959
Pseudogenes	163
Pseudogenes (frameshifted)	82
Protein encoding genes	2810
rRNA	6
tRNA	49
ncRNAs	3
Accession no.	NZ_QWLM00000000

**Table 3:** Predicted function of genes detected in the phenazine biosynthetic gene cluster (Phz BGC) of *D. abyssi* MT1.1^T^ (antiSMASH 5.1.0 and GenBank).

No.	Locus Tag	Nucleotide (nt)	Protein	Accession Number	Function	Homologue	Accession Number	Identity	Coverage Percentage	E Value
2	D1832_RS13610	846	PhzF family phenazine biosynthesis isomerase	WP_118914810	DHHA isomerase	PhzF family phenazine biosynthesis isomerase [*Streptomyces purpureus*]	WP_019884027	76.95%	99%	6e-150
3	D1832_RS15170	669	Hypothetical protein	WP_147362718	Unknown activity	Hypothetical protein D1832_13610 [*Dermacoccus abyssi*]	RHW43994	99.28%	62%	1e-98
4	D1832_RS13620	1125	3-deoxy-7-phosphoheptulonate synthase (PhzC)	WP_118914924	DAHP synthase	3-deoxy-7-phosphoheptulonate synthase [*Streptomyces* sp. uw30]	WP_147995366	67.65%	99%	6e-176
5	D1832_RS13625	1896	Phenazine-specific anthranilate synthase (PhzE)	WP_118914814	ADIC synthase	Phenazine-specific anthranilate synthase component I [*Streptomyces purpureus*]	WP_019884038	69.54%	98%	0.0
6	D1832_RS13630	306	Hypothetical protein (biosynthetic-additional thio_amide)	WP_118914815	Oxidoreductase	YdhR family protein [*Streptomyces* sp. uw30]	WP_147993498	62.11%	93%	9e-33
7	D1832_RS13635	621	Pyridoxamine 5’-phosphate oxidase (PhzG)	WP_118914817	Phenazine-1,6-dicarboxylic acid (PDC) formation	Pyridoxal 5’-phosphate synthase [*Streptomyces* sp. uw30]	WP_147993497	60.68%	100%	3e-78
8	D1832_RS13640	1842	Asparagine synthase (PhzH)	WP_118914818	Asparagine synthase (glutamine-hydrolyzing) activity	Asparagine synthase (Glutamine-hydrolyzing) [*Streptomyces thermocarboxydus*]	WP_137209574	79.13%	99%	0.0
9	D1832_RS13645	444	Phenazine biosynthesis protein: phzB	WP_118914868	6-amino-5-oxocyclohex-2-ene-1-carboxylic acid (AOCHC) dimerization	phenazine biosynthesis protein [*Streptomyces thermocarboxydus*]	WP_137209661	83.67%	100%	3e-91
10	D1832_RS13650	993	WYL domain-containing protein	WP_118914820	Ligand-binding regulatory domain	WYL domain-containing protein [*Streptomyces glaucescens*]	WP_043504928	69.78%	97%	2e-145
11	D1832_RS13655	402	RidA family protein	WP_118914821	Enamine/imine deaminase activity	RidA family protein [*Streptomyces exfoliatus*]	WP_037644077	80.30%	99%	2e-71
12	D1832_RS13660	492	DUF488 domain-containing protein	WP_118914870	Unknown	DUF488 domain-containing protein [*Streptosporangiaceae* bacterium YIM 75507]	WP_119930667	68.12%	98%	5e-70
13	D1832_RS13665	393	DUF4186 domain-containing protein	WP_118914823	Unknown	DUF4186 domain-containing protein [*Nocardia suismassiliense*]	WP_107655452	81.15%	93%	2e-66
14	D1832_RS13670	1617	Hypothetical protein	WP_118914825	Asparaginase activity	Asparaginase [*Blastococcus* sp. DSM 44205]	SDE71699	61.57%	100%	0.0
15	D1832_RS13675	344	TraR/DksA family transcriptional regulator	WP_118913125	Transcriptional regulation	DNA-binding protein [*Micropruina glycogenica*]	WP_105185941	62.92%	77%	2e-24
16	D1832_RS13680	360	VOC family protein	WP_118912933	Dioxygenase and glyoxalase	VOC family protein [*Propionibacterium freudenreichii*]	WP_013161949	92.50%	100%	2e-76
17	D1832_RS13685	309	TetR/AcrR family transcriptional regulator	WP_118914637	Transcriptional regulation	TetR/AcrR family transcriptional regulator [*Streptomyces mirabilis*]	WP_075033331	73.53%	99%	1e-41
18	D1832_RS13690	702	Alpha/beta hydrolase	WP_118914826	Carboxylic ester hydrolase activity (esterase)	Alpha/beta hydrolase [*Streptacidiphilus jeojiense*]	WP_030267495	47.84%	98%	2e-64
19	D1832_RS13695	195	Hypothetical protein	WP_147362719	Unknown	Hypothetical protein D1832_13690 [*Dermacoccus abyssi*]	RHW44004	98.44%	100%	2e-36
20	D1832_RS13700	579	TetR/AcrR family transcriptional regulator	WP_118914830	Transcriptional regulator	TetR family transcriptional regulator [*Actinotalea* sp. HO-Ch2]	WP_149205434	58.19%	91%	1e-54

**Table 4:** Additional proteins implicated in biosynthesis of dermacozines identified in the genome of *D. abyssi* MT1.1^T^.

Protein	AA	Accession Number	Function
Shikimate kinase	213	WP_118912523	Shikimic acid biosynthesis
Dehydroquinate synthase	354	WP_118912522	Shikimic acid biosynthesis
Chorismate synthase	407	WP_118912574	Shikimic acid biosynthesis
Enoyl-CoA hydratase	265	WP_118912167	β-hydroxyphenyl propionyl-CoA biosynthesis
3-hydroxyacyl-CoA dehydrogenase	704	WP_118913237	β-ketophenylpropionyl-CoA biosynthesis
Thiolase family protein	405	WP_047311533	Benzoyl-CoA biosynthesis
Pyridoxal phosphate-dependent aminotransferase	405	WP_047311611	Transamination
Acyltransferase family protein	667	WP_118912475	Acyl condensation
Class I SAM-dependent DNA methyltransferase	912	WP_118914833	Methylation

## Supplementary Materials

The following are available online at https://www.mdpi.com/1660-3397/18/3/131/s1, Figures S1–S9: Structure, LC MS and NMR spectra of dermacozine M (**11**) in DMSO-*d_6_*, Figure S10: ACD Labs ^13^C chemical shift prediction of **11**, Table S1: Putative cold shock and osmotic stress response genes, Table S2: Oxidative stress response and respiration-related genes, Table S3: Genes responsible for cell wall/membrane alteration and carbon starvation, Table S4: Putative organic matter-hydrolyzing enzymes.
