# Supplementary material for: Whole Genome Sequence of Dermacoccus abyssi MT1.1 Isolated from the Challenger Deep of the Mariana Trench Reveals Phenazine Biosynthesis Locus and Environmental Adaptation Factors
Source: Mar Drugs. 2020 Feb 25;18(3):131. doi: 10.3390/md18030131 (PMC7143476; doi:10.3390/md18030131)
Supplement: Supplementary file 1 [file marinedrugs-18-00131-s001.pdf]

Whole Genome Sequence of *Dermacoccus abyssi* MT1.1 Isolated from the Challenger Deep of the Mariana Trench Reveals Phenazine Biosynthesis Locus and Environmental Adaptation Factors

Wael M. Abdel-Mageed [1, 2,*], Bertalan Juhasz [3], Burhan Lehri [4], Ali S. Alqahtani [1], Imen Nouioui [5], Dawrin Pech-Puch [6], Jioji N. Tabudravu [7], Michael Goodfellow [5], Jaime Rodríguez [6], Marcel Jaspars [3,*] and Andrey V. Karlyshev [4]

[1] Department of Pharmacognosy, College of Pharmacy, King Saud University, P.O. Box 2457, Riyadh 11451, Saudi Arabia; wabdelmageed@ksu.edu.sa (W.M.A.-M.); alalqahtani@ksu.edu.sa (A.A.)

[2] Department of Pharmacognosy, Faculty of Pharmacy, Assiut University, Assiut 71526, Egypt

[3] Marine Biodiscovery Centre, Department of Chemistry, University of Aberdeen, Old Aberdeen, Scotland, AB24 3UE, UK; r01bj16@abdn.ac.uk

[4] School of Life Sciences Pharmacy and Chemistry, Faculty of Science, Engineering and Computing, Kingston University, Kingston upon Thames, Penrhyn Road, KT1 2EE, UK; b.lehri@hotmail.co.uk (B.L.); a.karlyshev@kingston.ac.uk (A.V.K.)

[5] School of Natural and Environmental Sciences, Newcastle University, Newcastle upon Tyne, NE1 7RU, UK; Imen.Nouioui@newcastle.ac.uk (I.N.); Michael.Goodfellow@newcastle.ac.uk (M.G.)

[6] Centro de Investigacións Científicas Avanzadas (CICA) e Departamento de Química, Facultade de Ciencias, Universidade da Coruña, 15071 A Coruña, Spain; dawrin.j.pech@udc.es (D.P.-P.); jaime.rodriguez@udc.es (J.R.)

[7] School of Forensic and Applied Sciences, Faculty of Science and Technology, University of Central Lancashire, PR1 2HE, Preston, UK.; jtabudravu@uclan.ac.uk (J.N.T.)

* Correspondence: wabdelmageed@ksu.edu.sa (W.M.A.-M.); m.jaspars@abdn.ac.uk (M.J.); Tel.: +96-654-3522-148 (W.M.A.-M.); Tel.: +44-122-4272-895 (M.J.)

Abstract

Dermacoccus abyssi strain MT1.1^T is a piezotolerant actinobacterium that was isolated from Mariana Trench sediment collected at a depth of 10898 m. The organism was found to produce ten dermacozines (A–J) that belonged to a new phenazine family and which displayed various biological activities such as radical scavenging and cytotoxicity. Here we report on the isolation and identification of a new dermacozine compound, dermacozine M, the chemical structure of which was determined using 1D and 2D-NMR, and high resolution MS. A whole genome sequence of the strain contained six secondary metabolite-biosynthetic

Content **Page**

Figure S1. Dermacozine M Atoms Numbering 04
Figure S2. HMBC and COSY correlations of dermacozine M 04
Figure S3. LC MS data and formula of dermacozine M (Orbitrap, HRESMIS) 05
Figure S4. ^{1}H NMR of dermacozine M (DMSO-d_6, 600 MHz) 05
Figure S5. HSQC spectrum of dermacozine M (DMSO-d_6, 600 MHz) 06
Figure S6. HMBC (7 Hz) spectrum of dermacozine M (DMSO-d_6, 600 MHz) 06
Figure S7. HMBC (2 Hz) spectrum of dermacozine M (DMSO-d_6, 600 MHz) 07
Figure S8. DQF-COSY Spectrum of dermacozine M (DMSO-d_6, 600 MHz) 07
Figure S9. NOESY spectrum of dermacozine M (DMSO-d_6, 600 MHz) 08
Figure S10. ACD Labs ^{13}C chemical shift prediction 08
Table S1. Putative cold shock and osmotic stress response genes. 09
Table S2. Oxidative stress response and respiration-related genes 10
Table S3. Genes responsible for cell wall/membrane alteration and carbon starvation 11
Table S4. Putative organic matter-hydrolyzing enzymes 12

gene clusters (BGCs), including one responsible for the biosynthesis of a family of phenazine compounds. A pathway leading to the biosynthesis of dermacozines is proposed. Bioinformatic analyses of key stress-related genes provide an insight into how the organism adapted to the environmental conditions that prevail in the deep-sea.

Keywords

Mariana Trench; *Dermacoccus* strain MT1.1^{T}; Dermacozines; Genome sequencing; Biosynthetic gene clusters

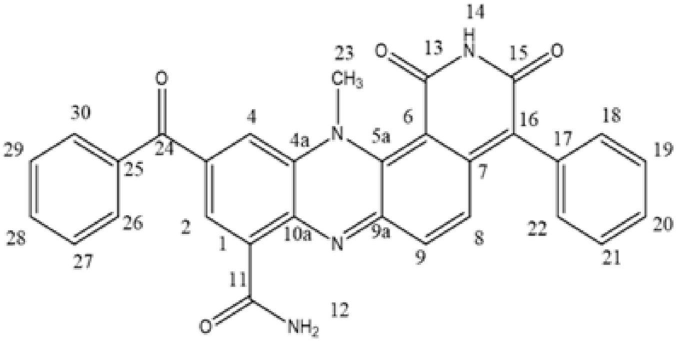

Fig. S1. Dermacozine M Atoms Numbering

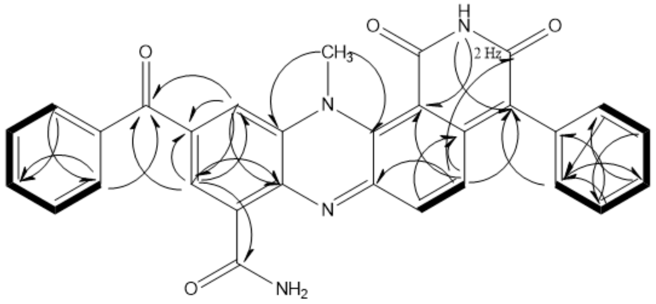

Fig. S2. HMBC and COSY correlations of Dermacozine M

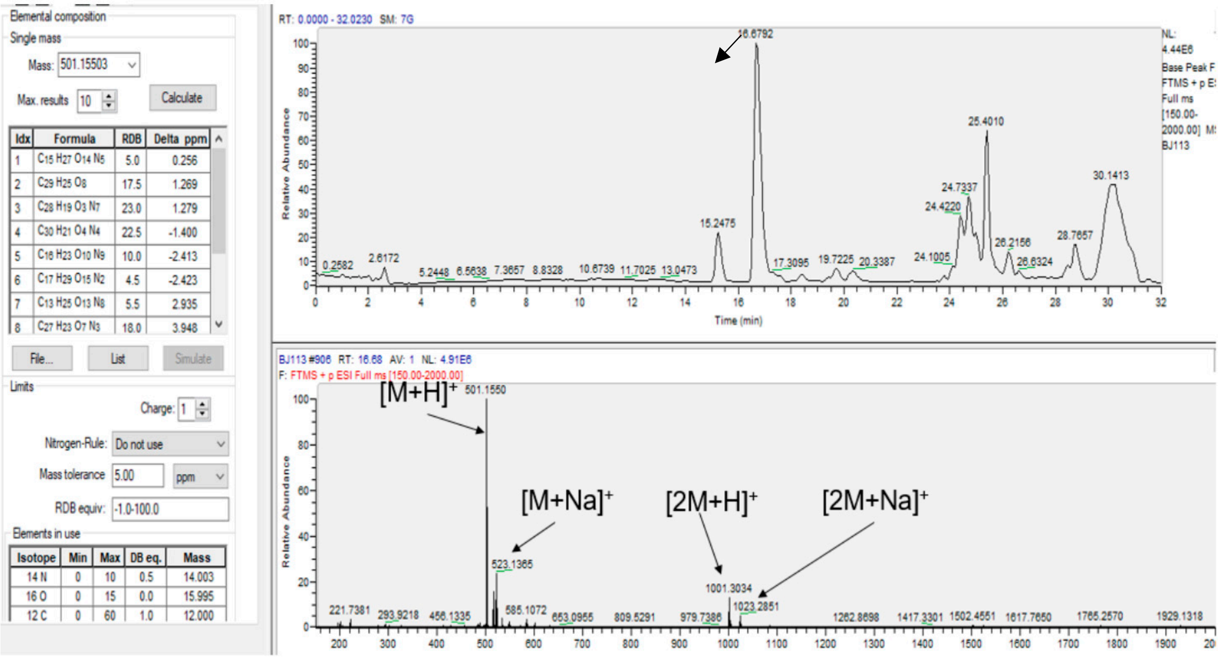

Fig. S3. LC MS data and formula of Dermacozine M (Orbitrap, HRESMIS)

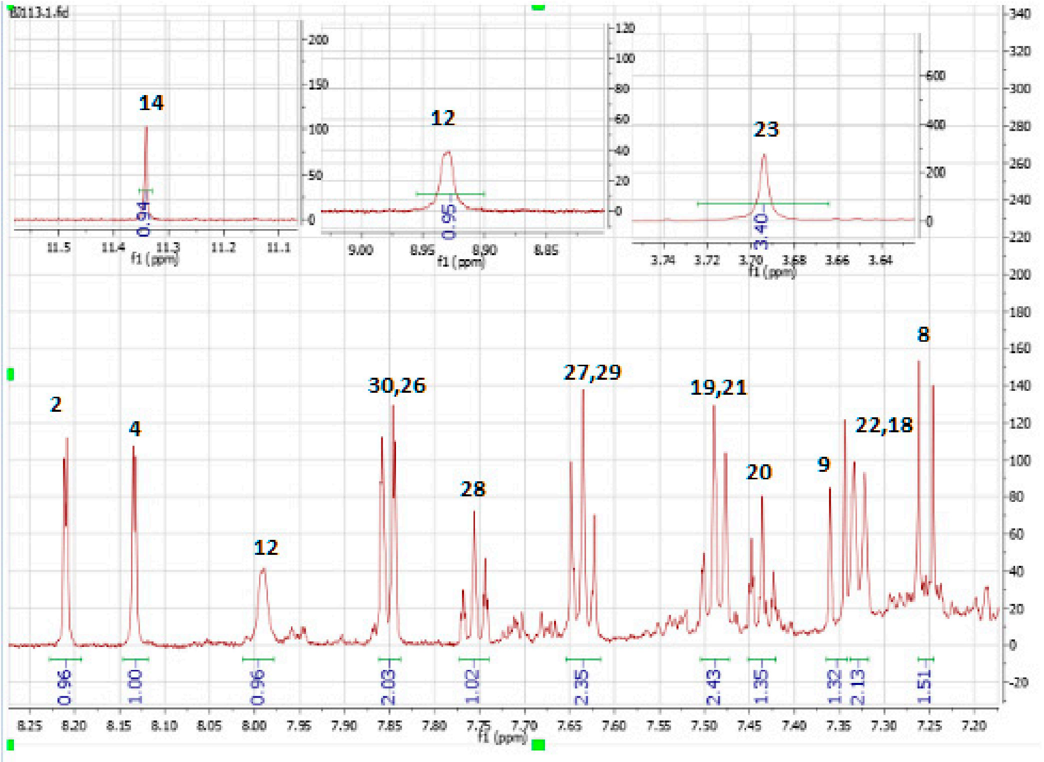

Fig. S4. **^{1}H NMR of Dermacozine M (DMSO-*d*$_6$, 600 MHz)**

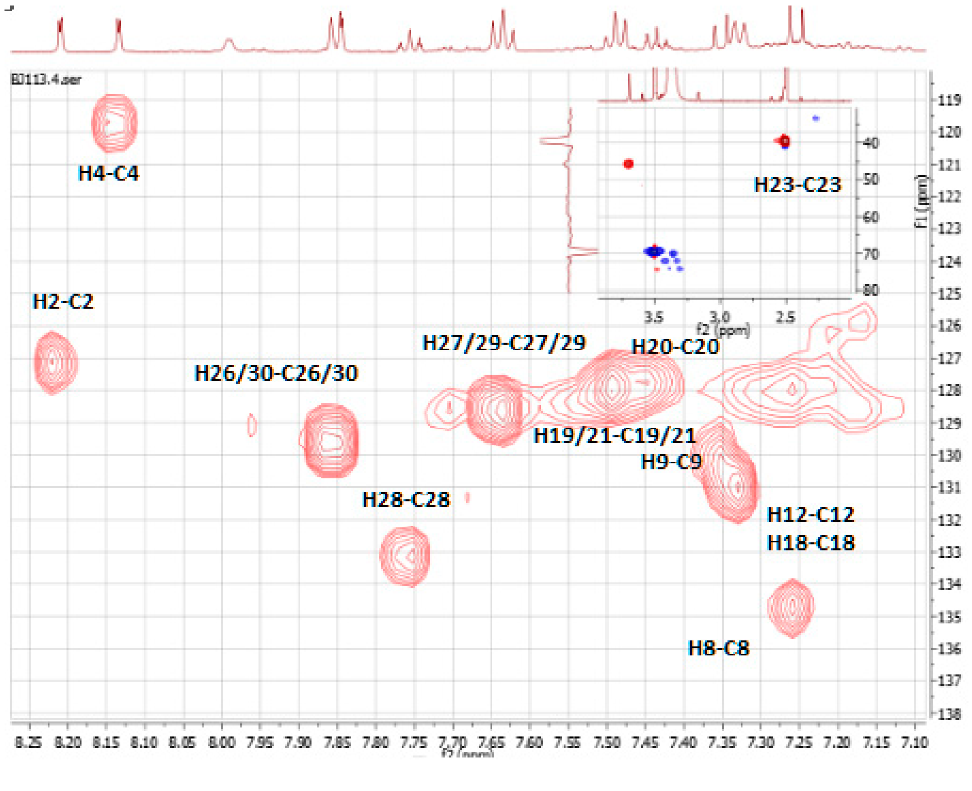

Fig. S5. HSQC spectrum of Dermacozine M (DMSO-*d*$_6$, 600 MHz)

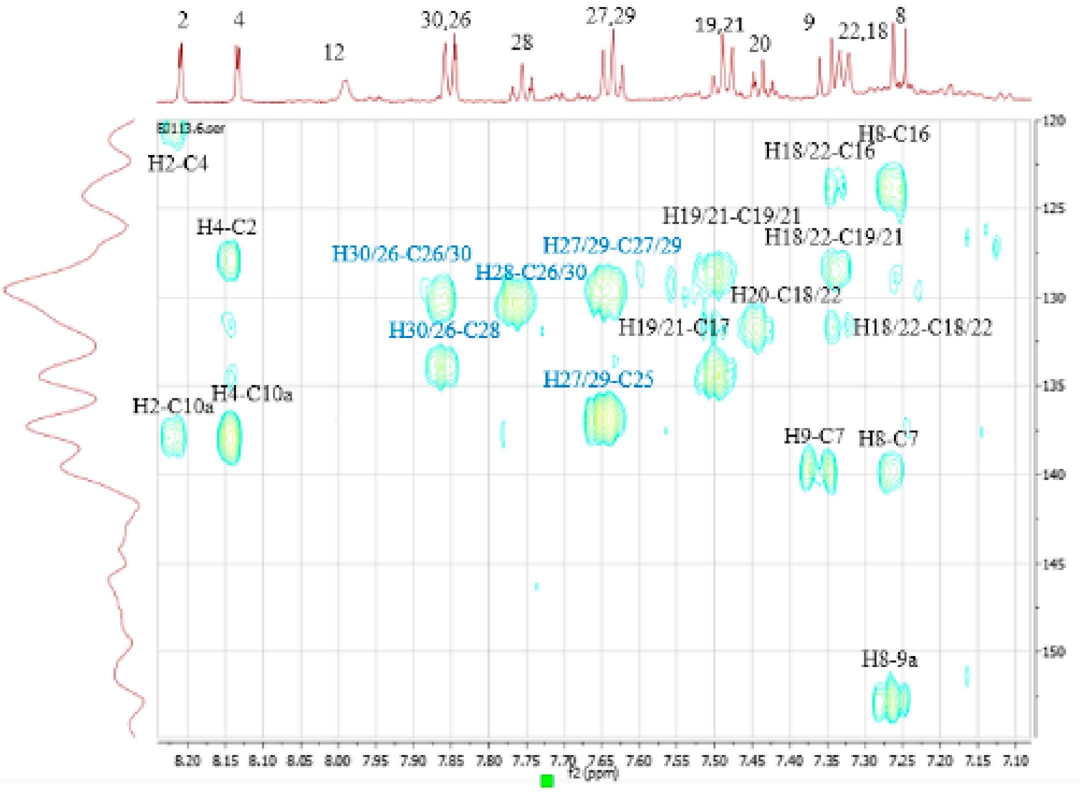

Fig. S6. HMBC (7 Hz) spectrum of Dermacozine M (DMSO-d_6, 600 MHz)

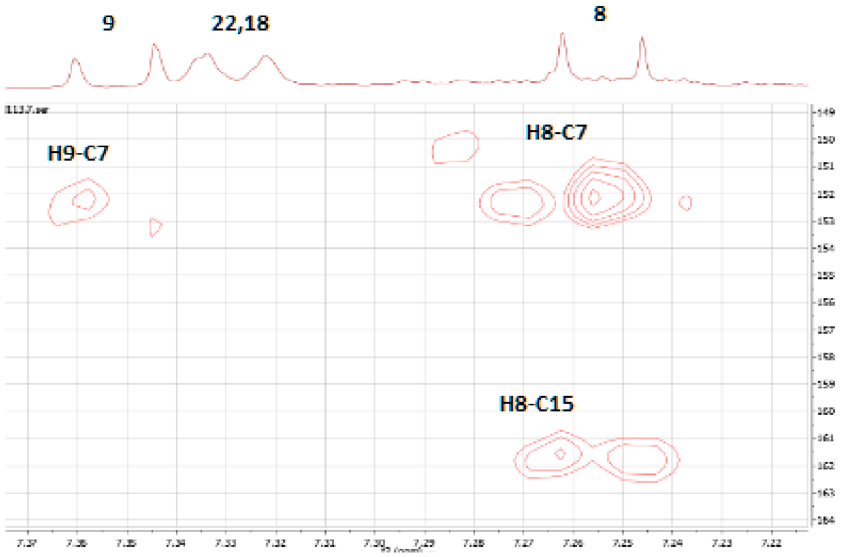

Fig. S7. HMBC (2 Hz) spectrum of Dermacozine M (DMSO-d_6, 600 MHz) showing H8-C15 correlation

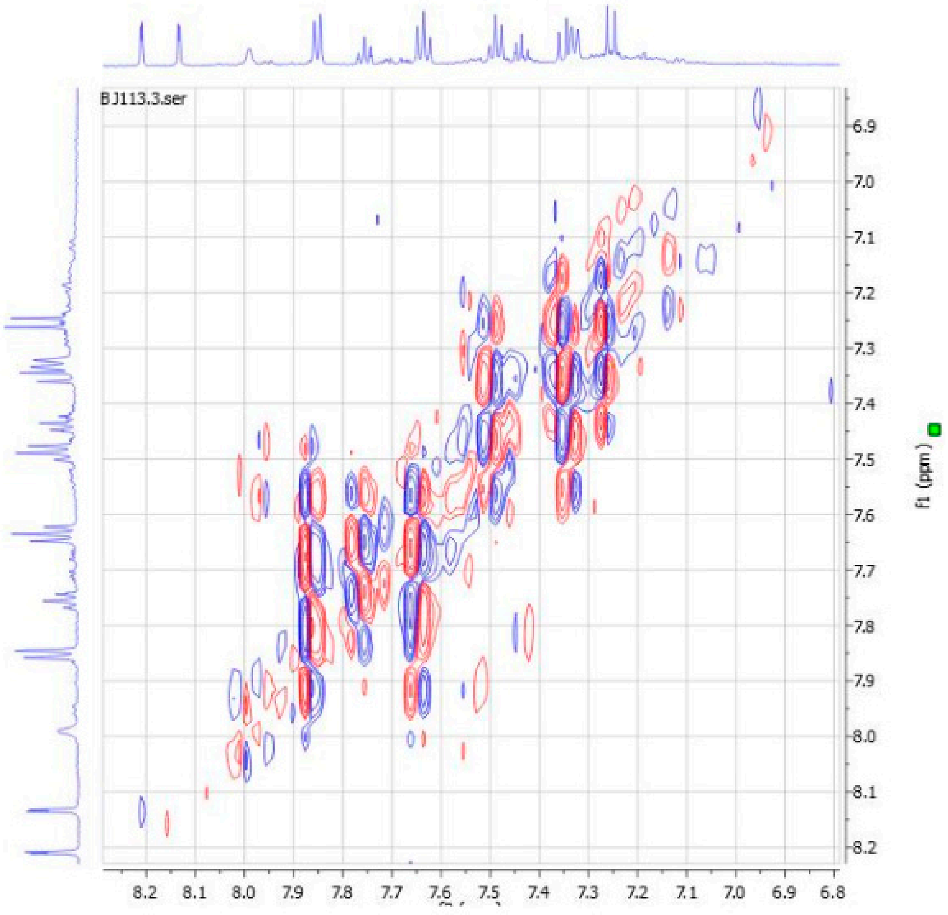

Fig. S8. DQF-COSY Spectrum of Dermacozine M (DMSO-d_6, 600 MHz)

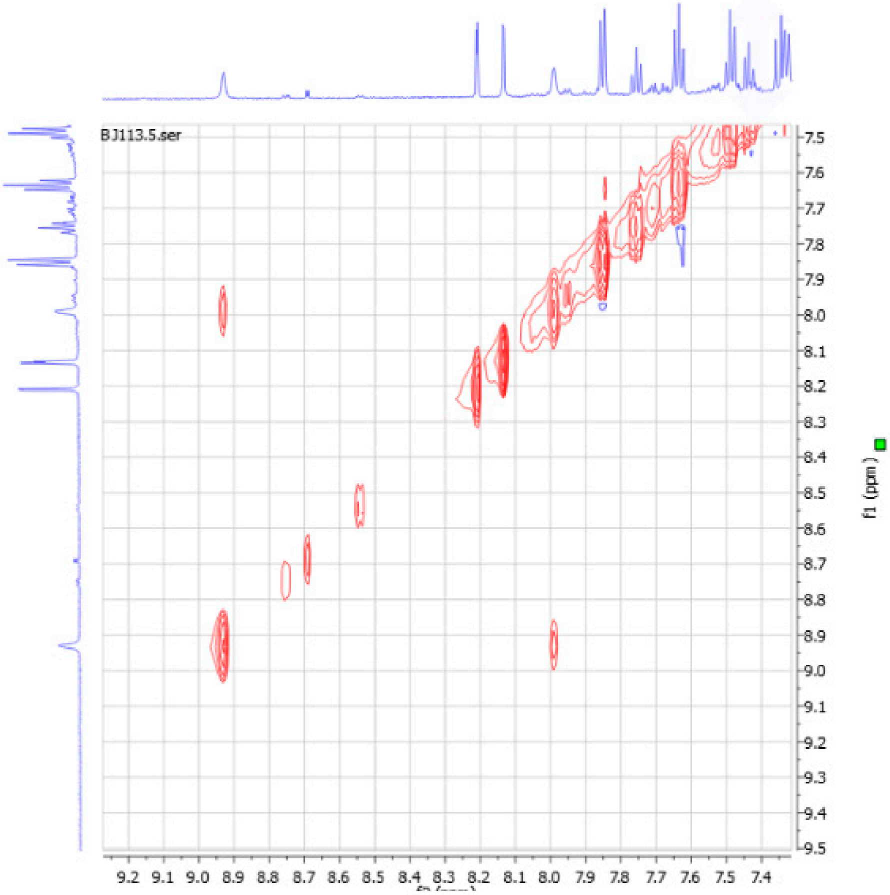

Fig. S9. NOESY spectrum of Dermacozine M showing correlation of the -NH protons of the carboxamide group (12) (DMSO-d_6, 600 MHz)

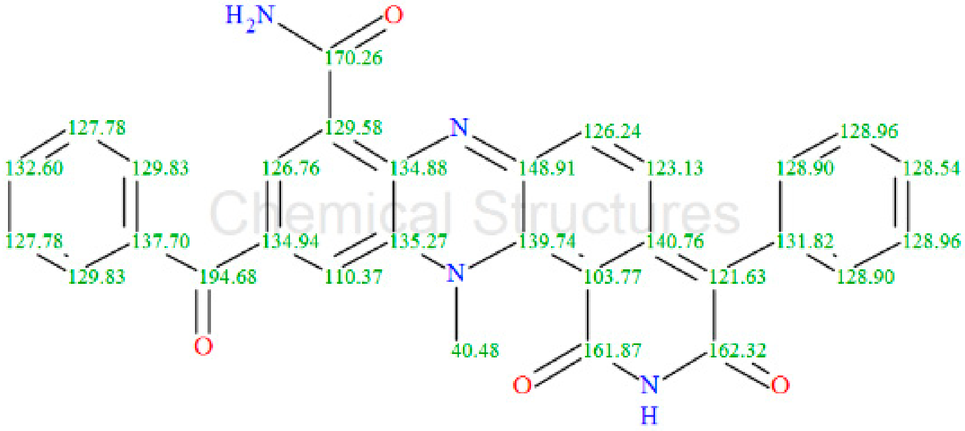

Fig. S10. ACD Labs ^{13}C chemical shift prediction simulating the missing C13 carbonyl chemical shift

Table S1. Putative cold shock and osmotic stress response genes identified in the genome of *D. abyssi* MT1.1^T.

Stress response	Protein	Gene	GenBank ID
**Cold Shock**	Cold-shock protein	*csps*	WP_118912295<br>WP_118912768
	Chaperonin GroEL	*groL*	WP_118913312<br>WP_118913486
	ATP-dependent chaperone ClpB	*clpB*	WP_118914595
	Co-chaperone GroES	*groES*	WP_118913311
	DEAD/DEAH box helicase	*deaD*	WP_118914699<br>WP_118913081
**Osmotic stress**	Choline dehydrogenase	*betA*	WP_118913227
	Betaine/Carnitine/Choline Transporter (BCCT family transporter)		WP_118912156<br>WP_118913228<br>WP_118913588
	Glycine/betaine ABC transporter substrate-binding protein	*opuA*	WP_118914940
	Aquaporin	*aqua*	WP_118912896
	Aspartate-semialdehyde dehydrogenase	*asd*	WP_118914342<br>WP_118914341
	Thiol reductant ABC exporter subunit CydC	*cydC*	WP_118912958
	Thiol reductant ABC exporter subunit CydD	*cydD*	WP_118912957
	Malate dehydrogenase	*mdh*	WP_118912226
	single-stranded DNA-binding protein	*ssb*	WP_118914057
	ABC transporter permease		WP_118912686<br>WP_118912989
	Metal ABC transporter permease		WP_118912641
	Amino acid ABC transporter permease		WP_118912632
	Branched-chain amino acid ABC transporter permease		WP_047310547
	Protein translocase subunit SecF	*secF*	WP_118913201
	Protein translocase subunit SecD	*secD*	WP_118913202

Table S2. Oxidative stress response and respiration-related genes in the genome of *D. abyssi* MT1.1^T.

**Stress response**	Protein	Gene	GenBank ID
**Oxidative stress**	Superoxide dismutase	*sodN*	WP_047310274
	Catalase	-	WP_118912840
	Thioredoxin	*trxA*	WP_118914664
	Thioredoxin-disulfide reductase	*trxB*	WP_118914013
	Peroxiredoxin	-	WP_047312091

	OsmC family peroxiredoxin	*osmC*	WP_118913166
	NAD(P)H-quinone oxidoreductase	-	WP_118914329
	Alkyl hydroperoxide reductase	-	WP_118912925
	Glutathione *S*-transferase family protein	-	WP_118913584
	Arsenate reductase	*arsC*	WP_118913813
	Glutaredoxin family protein	-	WP_118914221
**Respiration**			
	Cytochrome *d* ubiquinol oxidase subunit I	*cydA*	WP_118912949
	Cytochrome *d* ubiquinol oxidase subunit II	*cydB*	WP_118912950
	Cytochrome *c* oxidase subunit I	*ctad*	WP_118913146
	Cytochrome *c* oxidase subunit II	*coxb*	WP_118913145
	Cytochrome *c* oxidase subunit IV	-	WP_118913147
	cytochrome c oxidase assembly protein	-	WP_118912928
**Respiratory-related**	Arsenate reductase	*arsc*	WP_118913813
	Ferredoxin reductase	-	WP_118914278
	NADH-quinone oxidoreductase subunit A	*nuoA*	WP_118912274
	NADH-quinone oxidoreductase subunit B	*nuoB*	-
	NADH-quinone oxidoreductase subunit C	*nuoC*	WP_118912273
	NADH-quinone oxidoreductase subunit D	*nuoD*	WP_118915078
	NADH-quinone oxidoreductase subunit F	*nuoF*	-
	NADH-quinone oxidoreductase subunit H	*nuoH*	WP_118912336
	NADH-quinone oxidoreductase subunit I	*nuoI*	WP_082128608
	NADH-quinone oxidoreductase subunit J	*nuoJ*	WP_118912272
	NADH-quinone oxidoreductase subunit K	*nuoK*	WP_047310431
	NADH-quinone oxidoreductase subunit L	*nuoL*	WP_118912271
	NADH-quinone oxidoreductase subunit M	*nuoM*	WP_118912335
	NADH-quinone oxidoreductase subunit N	*nuoN*	-
	Glycerol-3-phosphate dehydrogenase		WP_118912897
	NAD(P)-dependent glycerol-3-phosphate dehydrogenase		WP_118912765
	L-lactate dehydrogenase	-	WP_118912189
	Formate dehydrogenase	-	WP_118912882
	Succinate dehydrogenase	-	WP_118913370
	Succinate dehydrogenase/fumarate reductase iron-sulfur subunit	-	WP_118913372
	Fumarate reductase/succinate dehydrogenase flavoprotein subunit	-	WP_118913371

Table S3. Genes responsible for cell wall/membrane alteration and carbon starvation in the genome of *D. abyssi* MT1.1^{T}.

Stress response	Protein	Gene	GenBank ID
**Membrane/cell wall alteration**	Beta-ketoacyl	*fabF*	WP_118912631
	3-oxoacyl-ACP reductase	*fabG*	WP_118912254
	Ketoacyl-ACP synthase III	*fabH*	WP_118912630
	enoyl	*fabI*	WP_118912407

	Enoyl-ACP reductase	-	WP_118912704
	Enoyl-CoA hydratase	-	WP_118912167
	Long-chain fatty acid-CoA ligase	-	WP_118912679<br>WP_118914883
	Acyl-CoA desaturase	-	WP_118913070
	Acyl dehydratase	-	WP_118912252
	3-hydroxyacyl-CoA dehydrogenase	-	WP_118913237
	Phytoene/squalene synthase family protein	-	WP_118912784
	UDP-N-acetylglucosamine 1-carboxyvinyltransferase	*murA*	WP_118913279
	Phosphopantetheinyl transferase	-	WP_118914698
**Carbon starvation**	Carbon starvation protein A	*csta*	WP_118913041
	Glycogen synthase	*glga*	WP_118912402
	Glycogen debranching enzyme	*glgx*	WP_118913222<br>WP_118914122
	Glycogen/starch/alpha-glucan phosphorylase	-	WP_118914411
	Carbonic anhydrase	-	WP_118912225<br>WP_118915142
	Carbon-nitrogen hydrolase family	-	WP_118912927

Table S4. Putative organic matter-hydrolyzing enzymes identified in the genome of *D. abyssi* MT1.1^T.

Enzymes	GenBank ID
**Protease**	
aminopeptidase P family protein	WP_118912327
aminopeptidase N	WP_118912267
M48 family peptidase	WP_118912322
M1 family peptidase	WP_118912863
M50 family peptidase	WP_118913143
S9 family peptidase	WP_118913024
alpha/beta hydrolase	WP_118912940
**Polysaccharase**	
Glycogen/starch/alpha-glucan phosphorylase	WP_118914411
beta-*N*-acetylhexosaminidase	WP_118913310
Polysaccharide deacetylase	WP_118914959
Polysaccharide deacetylase family protein	WP_118914361
Peptidoglycan endopeptidase	WP_118913505
alpha-amylase	WP_118914957
Carbohydrate kinase	WP_118912216
Glucosidase	WP_118913090
Galactokinase	WP_118912975

beta-*N*-acetylhexosaminidase	WP_118913310
**Purine catabolism**	
Purine-nucleoside phosphorylase	WP_118913393
hypoxanthine phosphoribosyltransferase	WP_118914540
**Allantoin**	
Allantoin permease	WP_118913617
Allantoate amidohydrolase	WP_118913007
